# RNAcare: integrating clinical data with transcriptomic evidence using rheumatoid arthritis as a case study

**DOI:** 10.1186/s12920-025-02162-z

**Published:** 2025-05-21

**Authors:** Mingcan Tang, William Haese-Hill, Fraser Morton, Carl Goodyear, Duncan Porter, Stefan Siebert, Thomas D. Otto

**Affiliations:** 1 School of Infection & Immunity, University of Glasgow, Glasgow, UK; 2 Research Software Engineering, MVLS SRF, University of Glasgow, Glasgow, UK; 3 LPHI, CNRS, INSERM, Université de Montpellier, Montpellier, France

**Keywords:** Gene expression analysis, Webserver, Patient clinic data, Data visualisation, Machine learning

## Abstract

**Background:**

Gene expression analysis is a crucial tool for uncovering the biological mechanisms that underlie differences between patient subgroups, offering insights that can inform clinical decisions. However, despite its potential, gene expression analysis remains challenging for clinicians due to the specialised skills required to access, integrate, and analyse large datasets. Existing tools primarily focus on RNA-Seq data analysis, providing user-friendly interfaces but often falling short in several critical areas: they typically do not integrate clinical data, lack support for patient-specific analyses, and offer limited flexibility in exploring relationships between gene expression and clinical outcomes in disease cohorts. Users, including clinicians with a general knowledge of transcriptomics, however, who may have limited programming experience, are increasingly seeking tools that go beyond traditional analysis. To overcome these issues, computational tools must incorporate advanced techniques, such as machine learning, to better understand how gene expression correlates with patient symptoms of interest.

**Results:**

Our RNAcare platform, addresses these limitations by offering an interactive and reproducible solution specifically designed for analysing transcriptomic data from patient samples in a clinical context. This enables researchers to directly integrate gene expression data with clinical features, perform exploratory data analysis, and identify patterns among patients with similar diseases. By enabling users to integrate transcriptomic and clinical data, and customise the target label, the platform facilitates the analysis of the relationships between gene expression and clinical symptoms like pain and fatigue. This allows users to generate hypotheses and illustrative visualisations/reports to support their research. As proof of concept, we use RNAcare to link inflammation-related genes to pain and fatigue in rheumatoid arthritis (RA) and detect signatures in the drug response group, confirming previous findings.

**Conclusion:**

We present a novel computational platform allowing the interpretation of clinical and transcriptomics data in real-time. The platform can be used for data generated by the user, such as the patient data presented here or using published datasets. The platform is available at https://rna-care.mvls.gla.ac.uk/, and its source code is https://github.com/sii-scRNA-Seq/RNAcare/.

**Supplementary Information:**

The online version contains supplementary material available at 10.1186/s12920-025-02162-z.

## Background

Autoimmune and autoinflammatory diseases incorporate a heterogeneous group of chronic diseases with substantial morbidity and mortality, thereby posing significant health challenges. In these conditions, immune dysfunction results in chronic inflammation and damage to various organs, including the joints (as seen in rheumatoid arthritis (RA)), the skin (as in psoriasis), and vital internal organs such as the heart and kidneys (as in systemic lupus erythematosus). Over time, autoimmune diseases can severely reduce quality of life, lead to long-term disability, and often require lifelong treatment to manage symptoms and prevent further damage. While there have been substantial advances in the treatment of RA and related rheumatic immune-mediated conditions based on an improved understanding of the underlying disease pathogenesis, there remains a significant unmet clinical need. Treatment responses remain unpredictable, and inadequate responses are common; some patients may respond to drugs with one mode of action, while others may respond to another drug or not at all. Currently, our ability to stratify (or personalise) treatment such that the right patient receives the right drug for their rheumatic disease is very limited [1]. Furthermore, despite improvement in inflammation levels with treatment, many patients with RA and similar immune conditions report persistent fatigue and pain despite treatment [2, 3]. However, multiple studies have shown discordance between physicians’ assessments of inflammation and patient-reported pain making the understanding of these immune diseases challenging.

One approach to study the underlying disease processes is to use transcriptomic data from relevant patient cohorts and correlate them with different phenotypes, like drug response/resistance, disease severity, disease mechanisms, or patient-reported symptoms, including pain and fatigue. Several studies have indicated that genes, transcripts and proteins associated with pain can be identified [4, 5], supporting the possibility that integrating transcriptomic and clinical data from well-phenotyped disease cohorts may provide further insights into disease mechanisms and patient outcomes. However, most analyses are performed by trained bioinformaticians who have relatively limited experience with the patients or diseases being studied, creating a data-analysis bottleneck, while clinicians often do not have the analytic skills to do this work themselves.

In response to this challenge, several tools have been developed to try and simplify transcriptomic analysis, especially through web-based platforms that streamline setup and configuration for users. The Shiny framework has facilitated the development of web interfaces for R-based pipelines, contributing to the growth of web applications for gene expression analysis [6–8]. Several computational tools exist to analyse transcriptomic data and visualise them through web frontends. However, most have limitations, see Supplemental Table 1: Phantasus [9], a web platform, integrates a highly interactive JavaScript heatmap interface with an R backend, supporting essential steps in gene expression analysis such as data loading, annotation, normalisation, clustering, differential expression (DE), and pathway analysis. However, Phantasus does not support transcriptomic dataset integration. Similarly, CFViSA [10] provides a comprehensive platform for omics-data visualisation and statistics, integrating microbiome and transcriptome analyses, but lacks support for batch integration and machine learning extensions. AmiCa [11], another web server for analysing microRNA and gene expression in cancer, does not accept user-uploaded data, while GEOexplorer [12] is limited to integration of two datasets, and does not include many options to visualise the batches. Notably, none of the aforementioned tools effectively integrate multiple cohorts of transcriptomic with clinical data.

In response to these limitations and the need to allow easy integration and analysis of transcriptomic datasets aligned with clinical phenotype in rheumatic disease cohorts, we present RNAcare, a web application designed for integrated gene expression and clinical data analysis. We first describe how our tool compares to commonly used existing tools and then report three case studies using RNAcare to perform transcriptomic analysis associated with key clinical parameters (pain, disease activity, fatigue and treatment response) in three RA patient cohorts.

## Implementation

**Fig. 1:**
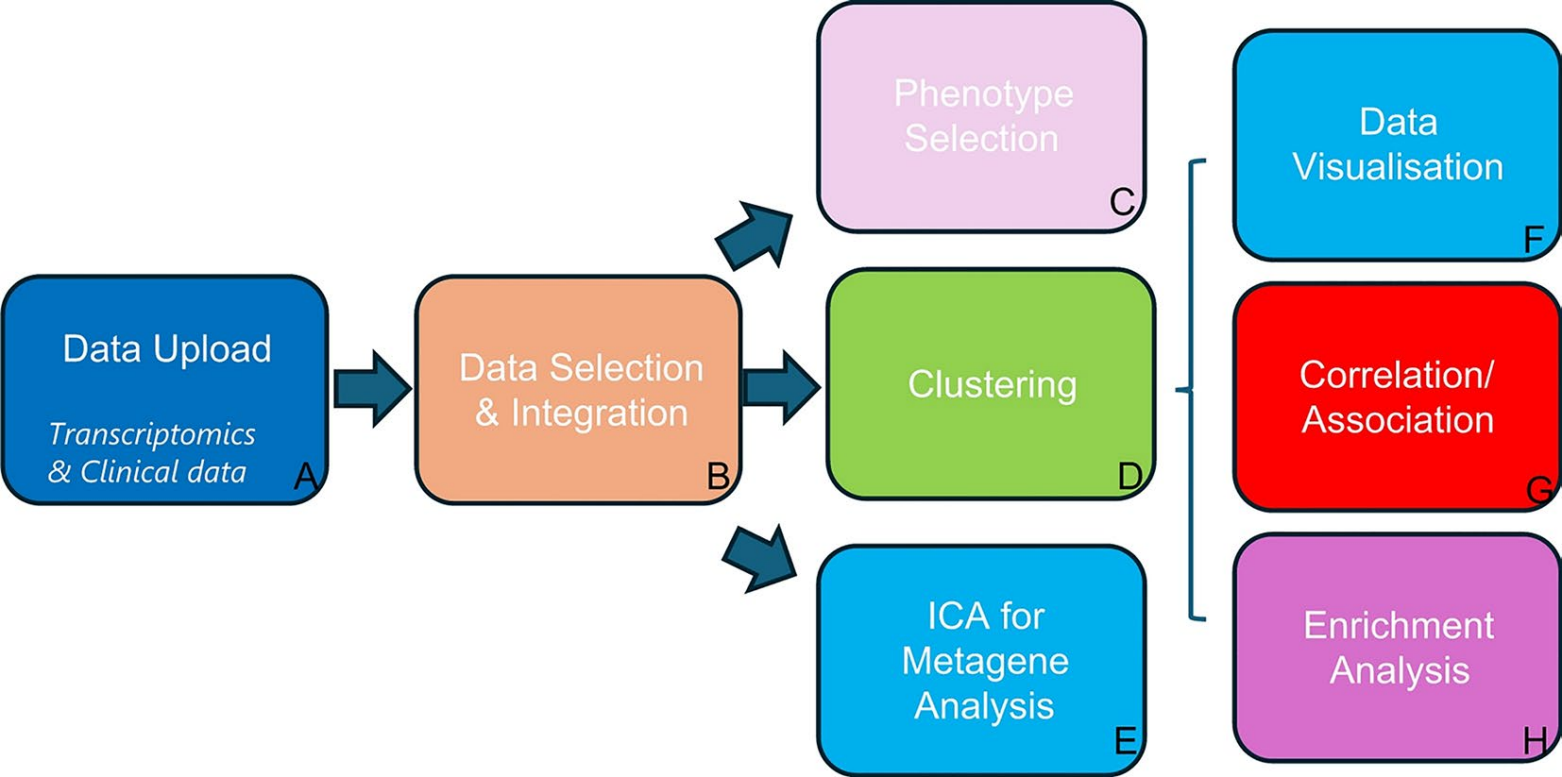
RNAcare workflow overview. (**A**) In the first tab, users can upload transcriptomics, proteomic and clinical data (optional); For proteomic data, some of the results from modules G and H may not be highly reliable due to the framework being primarily designed for transcriptomics. (**B**) In the Data Selection and Integration tab, users can combine different datasets and have the option to select various integration methods; (**C**) Phenotype Selection: new labels can be created and added from the clinical data (continuous or discontinuous) for downstream analysis; (**D**) Clustering: the transcriptomic data is visualised with cluster-based methods as well as projected into 2D; (**E**) Independent Component Analysis (ICA) for Metagene Analysis; (**F**) Data Visualisation for labels/clusters; (**G**) Correlation/Association to find relationships between transcriptomic data, clinical data and labels using Differential Expression or machine learning; (**H**) Enrichment Analysis for user’s cluster/label of interest

The overall aim of RNAcare is to provide a web-based tool simplifying the integration and analysis of transcriptomics and clinical data, showing the analysis workflow in tabs as outlined in Fig. 1 and described later in more detail. RNAcare was developed in Python, utilising several open-source packages. The web server was built using the Django framework, adhering to the FAIR (Findable, Accessible, Interoperable, and Reusable) principle [13], see Supplemental Fig. 1. The platform employs the Plotly graphics system for generating interactive visualisations on the fly. The platform can be installed locally from https://github.com/sii-scRNA-Seq/RNAcare/.

To enable several users working on the system simultaneously, Django is used to allow group and authentication management. System administrators can easily assign group roles to specific users, enabling them roles to only view and operate the permitted data. To improve the performance of the system we used Nginx server caches.

### Datasets

For testing RNAcare we used three RA datasets (Table 1)– Optimal management of RA patients requiring Biologic Therapy (ORBIT), Pathobiology of Early Arthritis Cohort (PEAC) and RA-MAP as part of the IMID-Bio-UK consortium.

**Table 1:** Overview of the PEAC, ORBIT and RA-MAP cohorts used in RNAcare. The clinical features are listed in supplemental Table 2

Cohort name	Number of participants	Data type	Tissue type	#Clinical feature
PEAC [14]	49	Bulk RNA-Seq	Blood	7
ORBIT [15]	139	Bulk RNA-Seq	Blood	16
RA-MAP [16]	63	Relative fluorescence units(RFU)	Plasma	12
	34	Microarray	Whole Blood	

The Pathobiology of Early Arthritis Cohort (PEAC) [14] was established with the aim to create an extensively phenotyped cohort of patients with early inflammatory arthritis, including RA, linked to detailed pathobiological data. The clinical and transcriptomic data can be found on EBI ArrayExpress with accession E-MTAB-6141. ORBIT [15] was a study comparing Rituximab to anti-TNF treatments. Blood was taken from patients before drug treatment. The RA-MAP Consortium [16] is a UK industry-academic collaboration to investigate clinical and biological predictors of disease outcome and treatment response in RA, using deep clinical and multi-omic phenotyping. The raw data were retrieved from accessions GSE97810 and GSE97948 on ArrayExpress. The clinical data can be found here: 10.6084/m9.figshare.c.5491611.v1.

#### Generation of input data

For the RA-MAP data (Array data/RFU), we used the provided normalised data. For the other two studies, we re-mapped the fastq files with hisat2 [17] (default parameters) and generated the read count matrix with featureCounts [18]. The clinical data were downloaded and included as text files in RNAcare.

#### Datasets for tool comparison

For comparison with other platforms, we used their example datasets. GSE53986 [19] for Phantasus, and GSE106382 [20] and GSE20589 [21] for GEOexplorer.

#### Clinical data used from the cohorts

RA disease activity was assessed using the validated DAS28 (Disease Activity Score using 28 joint counts) score, which was calculated from the original recorded 28 joint counts plus a blood marker of inflammation, typically the erythrocyte sedimentation rate (ESR) or the C-reactive protein (CRP) level [22]; Pain VAS is short for pain Visual Analog Scale (VAS) for self-reported pain, which is a unidimensional measure of general pain intensity, used to measure patients’ current pain level (no pain 0–4 mm; mild pain 5–44 mm; moderate pain 45–74 mm; severe pain 75–100 mm), which can be tracked over time, or used to compare pain severity between patients [23]. The pain VAS is not specific to RA and has been widely used in a range of patient populations, including those with other rheumatic diseases, patients with chronic pain, cancer, or even cases with allergic rhinitis [24]; The fatigue VAS ranging between 0 and 100 cm. Fatigue was considered mild if the fatigue VAS was < 20 cm, moderate if 20 ≤ VAS < 50 and severe if VAS ≥ 50 cm [25]. All of this clinical information is stored in CSV files (pain VAS for PEAC and ORBIT and fatigue VAS for RA-MAP) and, after curation, loaded into RNAcare.

### Data upload

The user has the option to use data uploaded previously, upload their data on the fly or a combination of both. As proof of concept, we included the three RA datasets described above, including clinical and expression data. RNA-Seq requires a read count matrix, which will be normalised later. Other omics data must be normalised initially before uploading. Clinical data are uploaded as a table.

### Data selection and integration

Before data integration, RNAcare detects whether the format of the expression data are integers or non-integers. For integers, the platform handles the expression data as RNA-Seq data (applies to PEAC and ORBIT), which are transformed from raw counts to count per million (CPM). For non-integers, for example microarray/proteomic data, the platform handles the expression data as normalised data (applies to RA-MAP), so users need to pre-normalize these data types.

The user has the option to log1p transform their data before harmonisation. RNA-Seq data is often highly skewed, with large differences in scale between genes. Log transformation helps stabilise the variance and makes the data more suitable for batch correction. If this option is selected, both the gene expression data and clinical numeric data will undergo log1p transformation. Conversely, if the log1p transformation is not selected, the gene expression data will remain unchanged, while clinical numeric data will be normalised to a 0–1 range.

For our case studies, we obtained the best integration results with the log1p normalisation. Therefore, for Case Study 1, we used log1p to integrate PEAC and ORBIT data; for Case Study 2, we used log1p to process the proteomics data but did not log1p transform the microarray data (because it had already been processed in the microarray data); for Case Study 3, we used log1p to transform the ORBIT data.

Next, the user has two options for the integration: Harmony [26] and Combat [27], and three options for feature reduction: principal component analysis (PCA) [28], t-distributed stochastic neighbour embedding (t-SNE) [29] and Uniform Manifold Approximation and Projection (UMAP) [30]. In general, it is unclear a priori which integration will generate the best data, and this will vary for different datasets, the user has different options for this in RNAcare [31].

### Phenotype selection

Users can define clinical classes, categorise continuous clinical parameters and generate new user-defined labels based on numerical variables from uploaded meta files (categorical variables in the meta files can be redefined for labelling). This feature allows for the creation of customised fields that can be used as targeted dependent variables in subsequent analyses.

### Clustering

Clustering allows grouping of patients by common expression patterns to facilitate the comparison of gene expression between different sample groups in order to identify differentially expressed genes (DEGs). We implemented various methods including K-means [32], Leiden [33], and HDBSCAN [34] to allow the user to choose their method of preference.

The default parameter for K-means is the number K, which needs to be set before running the clustering. The process will be terminated if users specify a very large K or the size of any of the clusters in the sample is less than 5. For Leiden, resolution is set to 1 by default. For HDBSCAN, the default parameter is minSize, representing the minimum size of clusters with a default value of 5 set in the interface.

Users can easily trace the clustering procedure by specifying the desired range and number of levels. The algorithm will automatically generate clusters based on the intervals within the specified range.

### ICA for metagene analysis

Independent component analysis (ICA) attempts to decompose a multivariate signal into independent non-Gaussian signals. Its application has already proved successful within a computational biology context [35]. We introduced ICA into transcriptomics to decompose the gene expression matrix into several independent components. Each independent component was treated as a metagene made up of explainable genes, characterised by a co-expression pattern, and was associated with certain meaningful biological pathways. In practice, we suggest that all clinical fields uploaded beforehand must be named as strings with prefix “c_”, such as “c_das”, which provides a convenient pattern for the program to recognise the expression data and apply the ICA algorithm.

### Data visualisation

Users can plot genes of interest based on clusters defined in earlier steps, with options to create violin plots, density plots, volcano plots, and heatmaps. The system can also suggest candidate genes through predefined algorithms.

### Correlation/Association

Here, the user has the option to associate selected clinical data parameters with the transcriptomic data in order to study phenotypes. Users can select the top N (default = 4) differential genes in each group after clustering. The platform can also apply the Lasso [36] and Ridge [37] methods to demonstrate feature importance. The default parameters for regularisation (Lasso and Ridge) are cv = 5 for 5 folds cross-validation, solver=”saga”, class_weight=”balanced” for potential imbalanced issues. Before regularisation, data needs to be standard scaled for either algorithm to compare all features equally. When we analyse the relationship between signatures and phenotype, Lasso is applied out of the consideration of feature reduction. When we analyse the relationship between metagenes and phenotype, Ridge is applied.

### Enrichment analysis

As the previous analysis will return gene lists of interest, this tab helps to identify biological pathways and gene sets significantly enriched in the dataset. Users can perform GO enrichment [38] analysis for specific clusters, enhancing their understanding of the underlying biological processes.

Another feature of RNAcare is allowing users to download all the data of the various analysis steps; results as CSV and figures as SVG, for further processing and use in publications.

## Results

Here, we present the first tool to our best knowledge that allows users without bioinformatics skills to integrate clinical and transcriptomics data. Although there are similar tools available (Supplemental Table 1), none has all the features of RNAcare. First, we compared it with two other tools to show that it has comparable performance. Further, we show RNAcare has more features in terms of data integration, contributor visualisation and DEGs for clustering before demonstrating three use cases.

### Comparison with selected existing tools

#### Phantasus

Phantasus is an analysis tool accessible via both web and local applications, designed to analyse transcriptome data derived from microarray or RNA-Seq technologies [9]. It does not allow the use of clinical data.

**Fig. 2:**
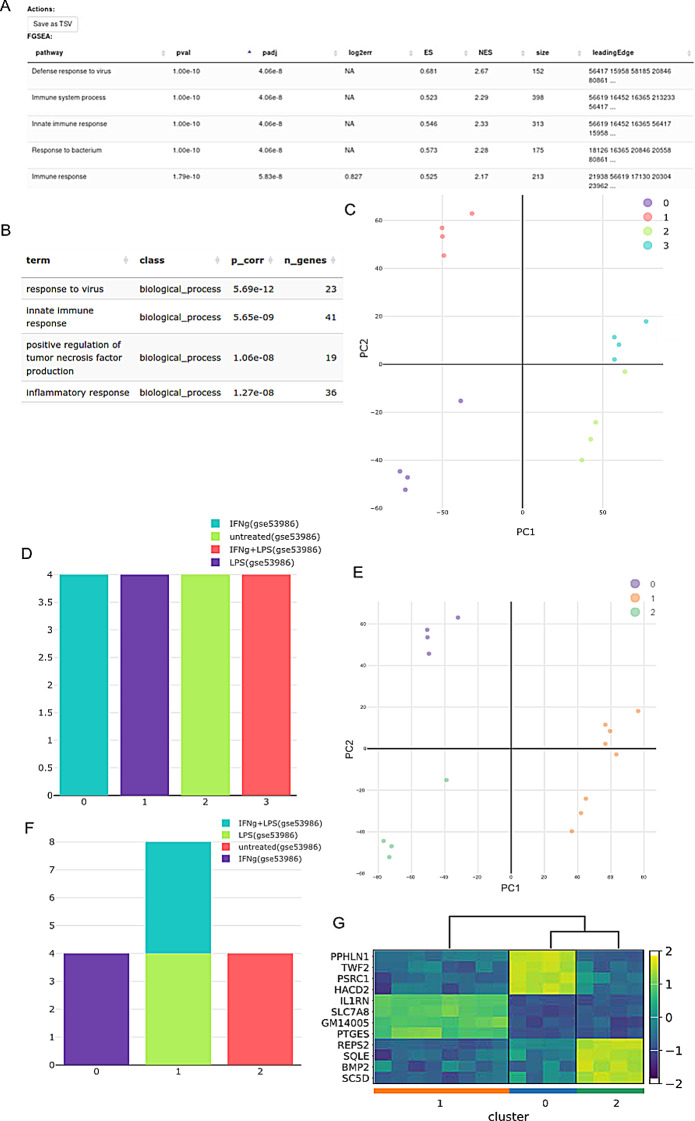
Comparison with Phantasus using GSE53986. (**A**) Go enrichment of LPS vs. untreated with Phantasus. (**B**) Go enrichment of LPS vs. all groups with RNAcare. (**C**) Visualisation for K means (K = 4) clustering using RNAcare, which Phantasus doesn’t support. (**D**) Contributions to the 4 clusters from each label. (**E**) Visualisation for K means (K = 3) clustering using RNAcare (**F**) Contributions to the 3 clusters from each label. (**G**) DEG heatmap plot for clusters with RNAcare

One dataset used in Phantasus consisted of 16 samples of bone marrow-derived macrophages, untreated and treated with three stimuli: lipopolysaccharide (LPS), interferon-gamma (IFNg), and combined LPS + IFNg (GSE53986). Both tools, Phantasus and RNAcare, have the option to perform differential gene expression and GO Enrichment. The results from GO enrichment are analogous (Fig. 2A, B).

The reason why Phantasus achieves such significant p adjusted values is because it only includes the top 12,000 expressed genes, and a binary DE between LPS and the untreated group was performed during the analysis. In RNAcare, we used all genes for analysis, which users can filter before uploading. Besides, we used global DEGs among all the four groups.

However, one useful feature of RNAcare missing in Phantasus is to visualise the clustering of the data. If we use K = 4 for clustering, we can see the data can be well clustered into 4 clusters matching with labels. It can be seen that the clusters are separated in the PCA plot visualisation (Fig. 2C). For each cluster we plot its contributors by Labels (Fig. 2D). We can also see IFNg + LPS and LPS are closed, so if we choose K = 3, we will see these two groups merge and find the common signatures compared to the other groups (Fig. 2E-G), as cluster 0 has samples from IFNg, cluster 1 samples from LPS and IFNg + LPS and cluster 2 primarily samples from the untreated group. These cluster visualisation methods are not part of Phantasus and are advantageous for RNAcare to allow users control in exploring their data.

#### GEOexplorer

Next, we compared GEOexplorer with RNAcare. We used two datasets for integration: (1) GSE106382, a dataset of induced pluripotent stem cells (iPSCs) from healthy controls, sporadic amyotrophic lateral sclerosis (ALS) and familial ALS patients including a subgroup of familial cases who carried a pathogenic mutation in the SOD1 gene (SOD1 ALS). We focussed on the SOD1 ALS samples and therefore will subsequently refer to these as model SOD1 spinal motor neurons. (2) GSE20589, which includes cervical spinal motor neurons from healthy controls and SOD1-related ALS post-mortem. These data are microarray data from the GPL570 platform.

Both tools allow similar analysis such as DE, GO enrichment and basic visualisations of the results (Supplemental Fig. 2A, B). However, GEOexplorer only supports integration of two datasets. In addition to allowing integration of multiple datasets, RNAcare also offers visualisation by batches to assess the quality of dataset merging after batch correction, making it well-suited for handling large-scale data integration. In contrast, GEOexplorer reviews batch-corrected results on a record-by-record basis (Supplemental Fig. 2C). Again, GEOexplorer does not have the function integrate clinical data.

Overall, RNAcare performs comparably to existing tools for standard analysis, however it has a new feature and an innovation of allowing the user to integrate clinical data. In the case studies we will introduce these features, however we first want to highlight the standard of implementation of RNAcare.

#### Computational advantages

Of note, RNAcare uses a more modern implementation, namely the RESTful Web Service architecture (Supplemental Fig. 3), which provides a flexible and standardised programmatic interface. This architecture facilitates seamless execution of stress tests, enabling the systematic evaluation of server performance under varying load conditions. In comparison, GEOexplorer and Phantasus are Shiny web apps, which inherently limits their programmability and scalability.

Although we have not tested this thoroughly in the other tools, RNAcare mitigates potential server abuse, with the evaluated system incorporating an average timeout mechanism of 180 s for machine learning tasks, ensuring that resource-intensive or malicious requests are automatically terminated upon exceeding the time limit. We stress-tested RNAcare with multiple users accessing it simultaneously, without any issues, in terms of processing and waiting times. For example, integrating and analysing two datasets (PEAC and ORBIT) on our server with 62 GB of RAM and 12 threads we observed no delays for up to 20 users, demonstrating the robustness of our system.

We next proceeded to test cases using RNAcare to associate clinical parameters of interest with combined transcriptional data in the uploaded RA cohorts.

### Case study 1: evaluation of the peripheral RNA signatures associated with pain in RA

Pain is an important clinical symptom in RA, which often persists, even when inflammation appears improved with immunomodulatory therapies, suggesting that pain in RA involves more than just inflammation. In this case study, we used two existing RA datasets (ORBIT and PEAC, baseline samples) which are pre-loaded in RNAcare, to find peripheral RNA markers associated with different levels of pain, measured using patient self-reported pain VAS [23]. The aim was to integrate the two datasets, perform different analyses to find the association of pain with different variables, and finally see if existing signatures could be detected in our datasets.

After importing the clinical and RNA-Seq data from ORBIT and PEAC into the RNAcare platform, we created a new categorical label to define two levels of pain, low and high. The pain VAS variable ranges from 0 to 5 (Supplemental Fig. 4). We set a threshold of 4 (log1p(45) ≈ 4), which divided the participants into two groups: no/low pain (low-53) and moderate/high pain (high-135). The advantage of RNAcare is that this label can now be used to perform further analysis, while all other derived labels and the original continuous pain feature were excluded from independent features in the subsequent modelling steps.

**Fig. 3:**
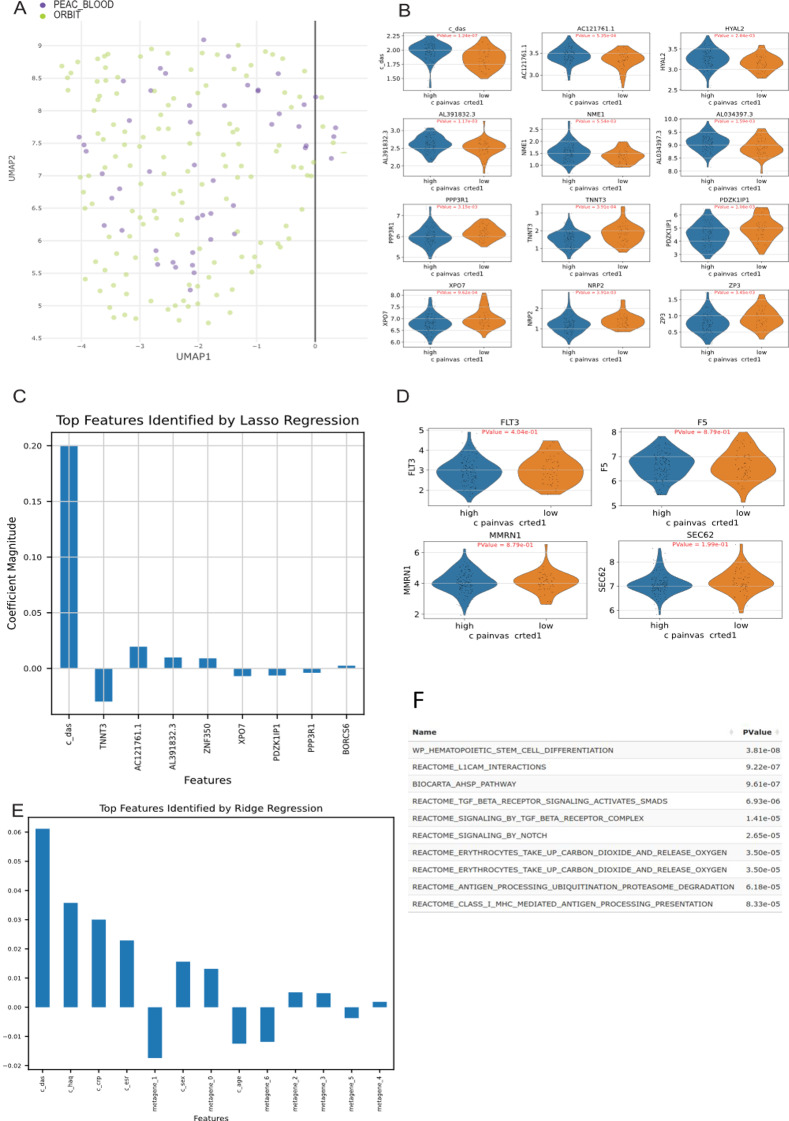
Case study 1: Data Integration for pain exploration. (**A**) Data integration settings and creation of customised labels visualised by UMAP after batch correction (**B**) Violin plot for DE genes of the labelled groups. (**C**) Lasso regression with cross-validation for the high pain group, significant genes are found for the created label. (**D**) Using RNAcare to visualise the previously identified markers from Hall et al. [5]. (**E**) Ridge regression with cross-validation for high pain group using ICA. Shown is metagene_1. (**F**) Pathway analysis for metagene_1

First, we integrated the data to account for batch effects. We applied the Combat algorithm which integrated the two datasets well, visible in the next tab of RNAcare with no dataset clustering on its own (Fig. 3A).

This successful integration allowed us to compare the features (gene expression and clinical data) between patients from low and high-pain RA groups. RNAcare uses Scanpy [39] in Python, which expects clinical features as genes. One difference compared to tools like DESeq2 is that Scanpy cannot regress out clinical features when doing DE analysis, and DESeq2 assumes that data follows the negative binominal distribution. During data processing, the platform runs a comparison automatically, finding the markers (downloadable from the EDA tab) as default candidates when the user wants to plot between different clusters (from the DGEA-Target Gene tab). In this case, we find that c_das (DAS score) is significant based on p adjusted value < 0.05 after the comparison. However, Fig. 3B still shows the top 12 biomarkers ordered by significance.

To take better advantage of the clinical data, we performed a Lasso regression to identify genes and clinical data associated with the different pain levels. This approach with the parameter class_weight=’balanced’ gives more weight to the patient group with a small number of participants while selecting only the most relevant and predictive features, ignoring irrelevant ones. The coefficient magnitude of every marker after Lasso regression with cross-validation for the high-pain group is plotted in Fig. 3C.

Comparing the results of the two methods, the first comparison only demonstrates the DAS score as a significant result, and the Lasso regression has a good overlap with other markers, though the order is different. The advantage of the Lasso integration is how it explains the signal. For example, the DAS score has the largest co-efficient (0.2), which contributes the most to the pain as expected. This suggests that increasing the log-transformed DAS score by 1 unit will result in a exp(0.2)-1 = 22.14% increase in the odds for patients to be in the high pain group, while controlling the other independent variables unchanged. A negative association is seen with TNNT3. TNNT3 encodes fast skeletal troponin T protein, and its GO annotation is actin binding. Two positively associated transcripts are AC121761.1 and AL391832.3 (a long non-coding RNA), are genes with limited available information.

Another feature of RNAcare is to check the expression of existing gene signatures in datasets. In order to see if the dorsal root ganglia (DRG) biomarkers identified by Hall et al. [5] were associated with pain in serum RNA we used RNAcare to visualise these biomarkers. As shown in Fig. 3D, those biomarkers show no significant difference in our datasets which are from PBMC.

In Fig. 3E, we used ICA, decomposing the expression data into seven metagenes. In this analysis, each metagene should represent biologically meaningful gene expression pattern of several genes that is statistically independent of other components. Next, we ran Ridge regression of those metagenes with the clinical features over the two pain groups. We can see that metagene_1 is mostly highly associated with high pain. Therefore, we performed a pathway analysis for the metagene_1 (Fig. 3F). Several significant terms returned, including L1CAM interactions and Notch signalling pathways, which have already been associated with pain in the tissue of DRGs, particularly in the context of neuropathic pain [40–42].

**Fig. 4:**
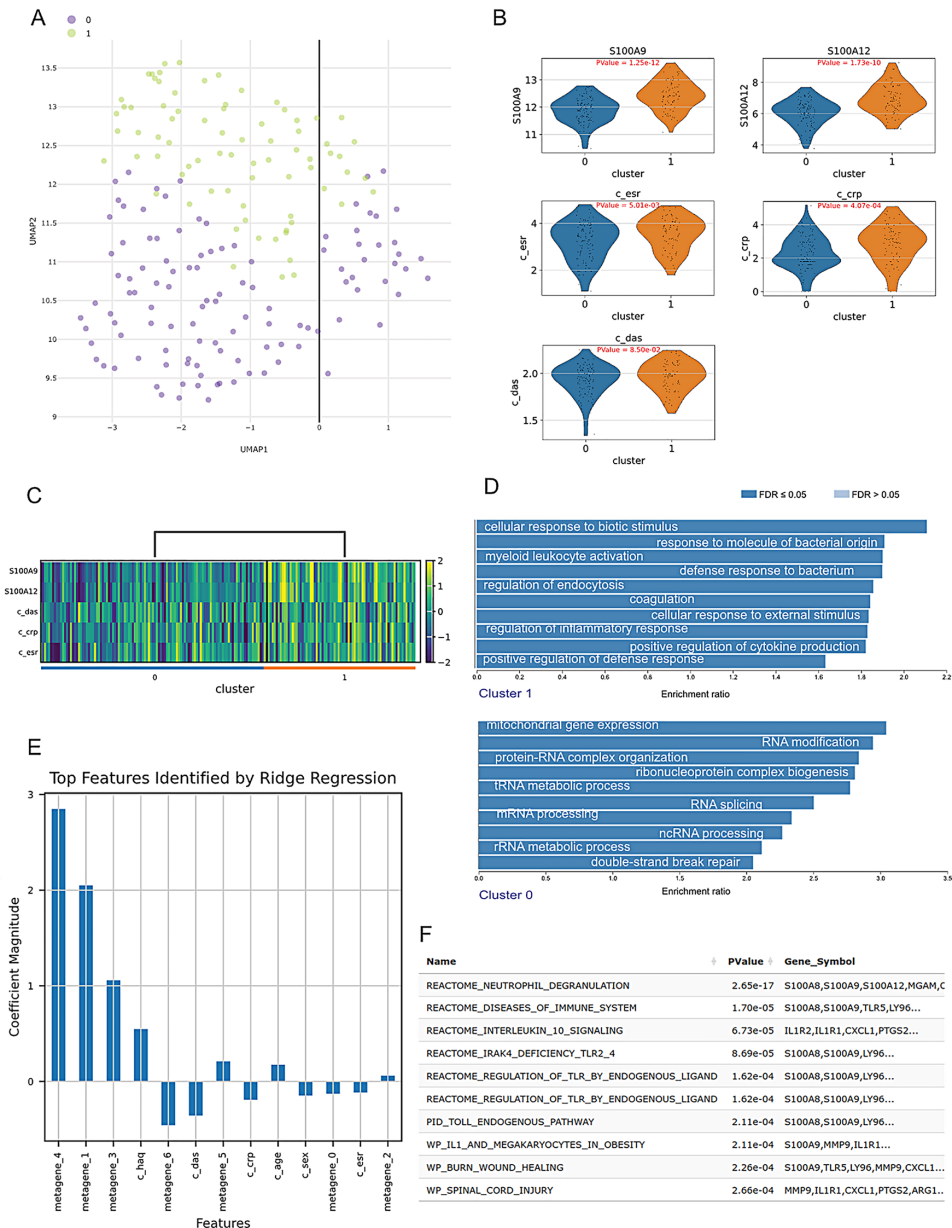
Exploring the K-means clustered (k = 2) data from Case Study 1. (**A**) Clustering the integrated data. (**B**) Violin plot for the clusters. GSEA for the clusters. (**C**) Heatmap plot for the clusters. (**D**) GSEA for the clusteers. (**E**) Top features identified by Ridge regression after applying ICA for cluster 1. (**F**) Pathway analysis for metagene_4

Another way to visualise the data in RNAcare is with clustering-based methods. To analyse transcripts correlated to disease activity, which are indirectly related to pain, we performed clustering of the two cohorts into two groups using K-Means with two clusters (Fig. 4A). The 3,200 downregulated and 286 upregulated significant markers (adj Pvalue < 0.05 and|logFC| > 0.5) are identified from cluster 1 (Supplemental Data File 1, as downloaded from RNAcare, unfiltered), revealing distinct functional roles. We did Gene Set Enrichment Analysis (GSEA) for the clusters (Fig. 4D): cluster 1 contains upregulated genes that are involved in inflammation and immune modulation. As expected, transcripts linked to the inflammatory response, such as S100A9, S100A12, and IL1R2, are occurring in the cluster.

RNAcare offers tools to visualise those features. Violin and heatmap plots of S100A9, S100A12, disease activity (DAS), CRP, and ESR showed differences between the two clusters (Fig. 4B, C).

To explore further the clinical features of cluster 1, we used original integrated data, processed it by ICA and ran Ridge regression on cluster 1 (Fig. 4E). RNAcare enabled us to perform pathway analysis of individual metagenes, in this case study the most significant one being metagene_4 (Fig. 4F). The top genes were S100A8, ILR1 and again S100A9.

Overall, we have shown in this case study that it is possible to use the standard pipeline of RNAcare with the pre-loaded data to perform different analyses and generate new hypotheses. Although a limited number of genes were differentially expressed in our analysis, we could not find novel insight to associate pain with signatures. However, we were able to replicate existing results. We have shown that it is possible to concatenate analyses, for example, combining ICA with clustering and pathway analysis, highlighting the dynamic functionalities of RNAcare. Limitations here might be due to the different nature of the two cohorts and the limited sample size. Finally, it is up to the user to ensure correction for multiple testing, when using several tools within RNAcare.

### Case study 2: evaluating the peripheral RNA signatures associated with fatigue in RA

Fatigue is a significant cause of poor quality of life in patients with RA and together with pain, is reported by patients as their most troublesome symptom [43]. The underlying pathophysiology of fatigue in RA, and other chronic immune conditions, remains incompletely understood. Tanaka et al. [44] proposed four key mechanistic themes for fatigue in RA, namely inflammation, hypothalamic-pituitary-adrenal axis, dysautonomia, and monoamines, which have a complex network of interconnections between themes, suggesting a key role for inflammatory cytokines in the development and persistence of fatigue.

In this example, we hypothesised that there are peripheral markers associated with fatigue severity. Similar to the first case, we first tried to find significant biomarkers associated with fatigue. However, we did that first at the proteomic level and then tested the significance of the biomarkers at the transcriptomic level. This sequential workflow allowed us to validate findings and evaluate whether the fatigue-associated signatures were conserved across the proteomic and transcriptomic layers. As for the dataset, we used the RA-MAP cohort, which included fatigue VAS.

**Fig. 5:**
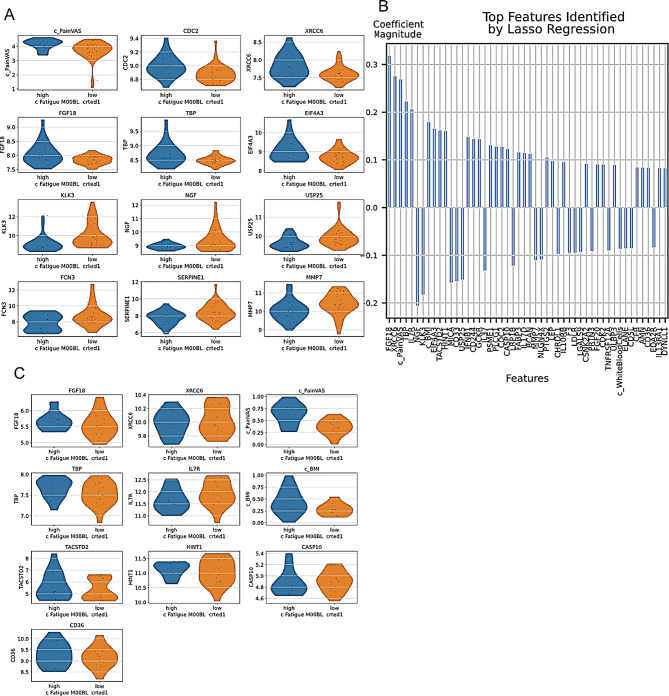
Case Study 2: Exploring fatigue with RNAcare. (**A**) Violin plot for features of interest in different groups to Fatigue. (**B**) Lasso regression with cross-validation to prioritise important features for the group with high fatigue scores. (**C**) Violin plot for corresponding features at transcriptomic level after running Lasso on Proteomics data

A customised label was created based on fatigue levels (threshold = log1p(50) ≈ 4), stratifying the proteomic samples into two distinct groups (Supplemental Fig. 4B). By assigning the label, we were able to explore DE between the “high” and “low” groups of 34 and 29 samples, respectively (Fig. 5A). However, there were no significant differences between the groups (all adjusted p values > 0.05). Therefore, we compared it with the result of Lasso and analysed the overlapping 8 markers (Fig. 5B): FGF18, XRCC6, TBP, NGF, KLK3, EIF4A3, USP25, CDC2.

To confirm the Lasso results, we uploaded the microarray data with the RA-MAP cohort, which yielded 34 records (14 high fatigue, 20 low fatigue, Fig. 5C). Although there is a trend confirming the Lasso result, none of the results were statistically significant.

In conclusion, we could not find any new associations, especially as many findings were above the significance cutoff. Therefore, we would argue that larger sample sizes are needed to obtain more confident results. At the same time, this highlights a common danger when performing a meta-analysis of finding some (few) interesting hits but not obtaining a clear significant signature of a biological process. However, importantly for this proof-of-principle paper, a powerful tool now exists to generate hypotheses that can be tested by clinicians once more data becomes available.

### Case study 3: evaluating peripheral baseline DEGs associated with treatment response

Resistance to drug treatment is a major problem in treatment responses of RA [45]. Approximately 30-40% of patients fail to respond to anti-TNF treatment. Although treatment response is likely to be multifactorial, some markers have been replicated consistently between studies [46].

To highlight the functionality of RNAcare, we used a subset of the ORBIT dataset, focusing on exploring the potential genes at baseline associated with responders and non-responders only for anti-TNF after 6 months of treatment. We grouped patients with remission and good response as the responder group (*N* = 35) and patients with partial/non-response as the non-responder group (*N* = 6) by using the metadata provided.

**Fig. 6:**
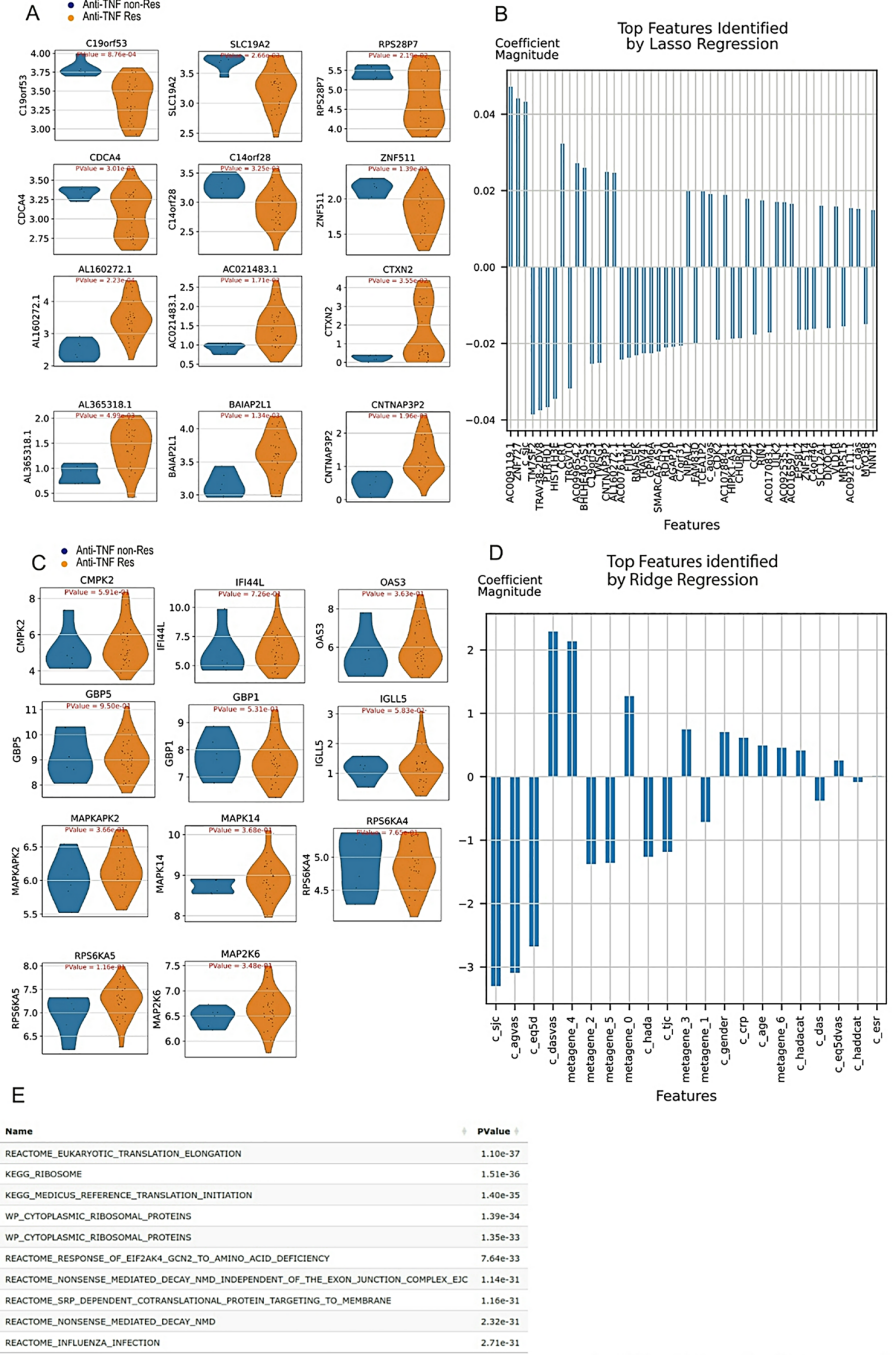
Case Study 3: Anti-TNF treatment comparison. (**A**) Violin plot of the two groups using Differential Expression. (**B**) Lasso regression with cross-validation for response group. (**C**) Gene verification for other literature. (**D**) Ridge regression with cross-validation for response group with metagenes. (**E**) Pathway analysis for metagene_4

We performed the same analysis pipeline as before, generating a DE of the data with Scanpy and the association with Lasso. Performing DE with a cut-off for corrected p-value of 0.05, we obtained two significantly upregulated DEGs, C19orf53 and SLC19A2, and two downregulated DEGs, CTXN2 and AC021483.1, for the non-responder group (Fig. 6A). After the intersection with the Lasso results (cut-off of 0.05, Fig. 6B), we were left with C19orf53, found in IFN cDNA libraries, producing the protein L10K, an interferon-stimulated gene (ISG) product still not well-characterised [47].

Previously, Yu et al. [48] showed that 41 response-associated genes in 82 RA patients shared common immune pathways including type I IFN signalling, and they reported six upregulated DEGs, namely CMPK2, IFI44L, OAS3, GBP5, GBP1, IGLL5, for non-response group and 305 DEGs for response and moderate response groups. Coulthard et al. [49] reported associated markers at proteomic level, including MAPKAPK2, MAPK14, RPS6KA4, RPS6KA5 and MAP2K6.

We took those gene lists and looked for differences in our datasets. Although we detected those transcripts (Fig. 6C), none of the results were statistically significantly different between the two groups, possibly as our sample size was very small.

Finally, in Fig. 6D, we used ICA, decomposing the expression data into seven metagenes and ran Ridge regression with clinical features for the non-response group. We can see metagene_4 is the most highly associated in the metagenes. It contains the genes HSP90AA1, many RPL and RPS (ribosomal genes). We performed a pathway analysis (Fig. 6E). As expected, we obtained terms enriched in translation and ribosomal signature due to the RPL and RPS genes. However, we could not find any novel insights to explain our treatment response.

## Conclusion

RNAcare has been designed to enable gene expression analysis of user-uploaded/built-in gene expression data with clinical data without the need to be proficient at programming or have advanced bioinformatics skills. It is available as a web server with three datasets, and it can be installed locally to allow the analysis of other datasets.

One limitation of web-based tools like RNAcare is that they can’t track how many analyses a user performs, making it difficult to account for multiple testing. Therefore, users should be aware of the risk of multiple testing and use RNAcare primarily as a tool for hypothesis generation. Any insights should be validated using independent datasets or experimental follow-up.

In our case, the datasets we chose did not yield any novel insights into pain, fatigue or drug treatment response; however, they highlighted future analysis opportunities. In contrast, currently available tools have limited ability to integrate clinical data with transcriptomics data. One of the reasons might be that, to date, few clinical parameters are included when submitting transcriptomics data to the repositories. However, we postulate that just by having those parameters, more integrative analysis can be performed, allowing us to leverage the existing data in more depth. At the same time, our case studies associating pain and fatigue with their transcriptomics data show potential avenues of analysis, albeit some of the results did not yield many significant results.

We hope that, due to recent developments in AI, more data will become available, and RNAcare will allow users without a bioinformatics background to integrate data to generate new hypotheses. Nevertheless, although we give clinicians the opportunity to analyse data themselves, it would be essential to work with data scientists to understand the nature of the data, the need for sufficient biological replicates, and discuss limitations in the generalisation of the findings, especially the highlighted issue of multiple testing. Together with this expertise and larger data sets, RNAcare will be a powerful tool, facilitating the generation of hypotheses that can be tested through follow-up experiments.

### Availability and requirements

Project name: RNAcare.

Project home page: https://github.com/sii-scRNA-Seq/RNAcare/.

Operating system(s): Platform independent, tested on Linux (Ubuntu).

Compatible browsers: Firefox/Chrome.

Programming language: Python, JavaScript.

Other requirements: Python > = 3.8.

License: ISC licence.

Any restrictions to use by non-academics: No restriction.

## Electronic supplementary material

Below is the link to the electronic supplementary material.

**Supplementary Material 1:** Supplemental data

**Supplementary Material 2:** Supplemental figures and tables

## Data Availability

The code of can be found https://github.com/sii-scRNA-Seq/RNAcare/ and the webserver at https://rna-care.mvls.gla.ac.uk/. All data used are from published sources, as indicated in the Implementation section, or was approved by appropriated review boards. Accession numbers are: E-MTAB-6141, GSE97810, GSE97948, GSE53986, GSE106382 and GSE20589.

## Abbreviations

RNAcare: RNA-based Clinical Analysis and Research Engine
FAIR: Findable, Accessible, Interoperable, and Reusable
EDA: Exploratory Data Analysis
DGEA: Differential Gene Expression Analysis
DE: Differential Expression
IMID: Immune-mediated inflammatory diseases
PEAC: Pathobiology of Early Arthritis Cohort
ORBIT: Optimal management of rheumatoid arthritis patients who Require Biological Treatment
CPM: Count Per Million
VAS: Visual Analogue Scale
BMI: Body Mass Index
DAS28: Disease Activity Score using 28 joint counts
PCA: Principal Component Analysis
t-SNE: t-Distributed Stochastic Neighbour Embedding
UMAP: Uniform Manifold Approximation and Projection
REST: Representational State Transfer
CSR: Class Switch Recombination
ESR: Erythrocyte Sedimentation Rate
