# Supplementary material for: RNAcare: integrating clinical data with transcriptomic evidence using rheumatoid arthritis as a case study
Source: BMC Med Genomics. 2025 May 21;18:93. doi: 10.1186/s12920-025-02162-z (PMC12096495; doi:10.1186/s12920-025-02162-z)
Supplement: Supplementary file 2 — Supplementary Material 2: Supplemental figures and tables [file 12920_2025_2162_MOESM2_ESM.pdf]

Supplemental Material:

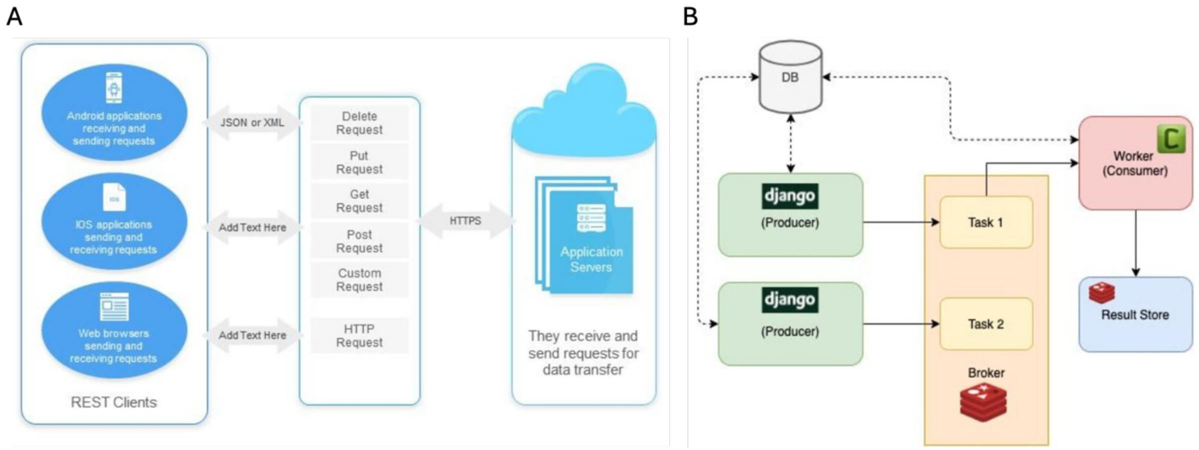

Supplemental Figure 1. Platform Architecture. **(A)** Django is used to establish a RESTful webservice; **(B)** At the backend, Celery is used to establish a distributed scheduling system for multiple users.

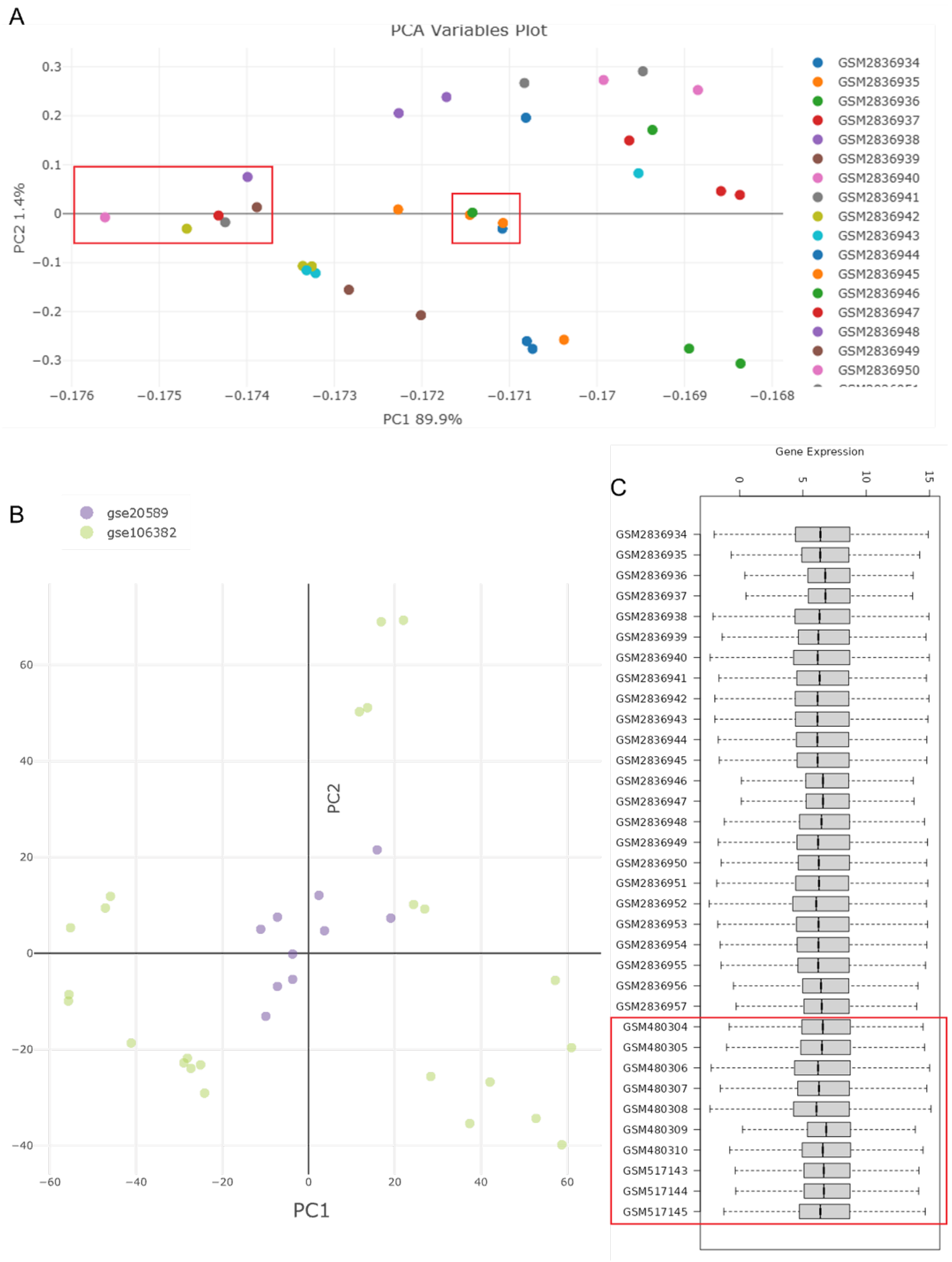

Supplemental Figure 2. Comparison with GEOexplorer using GSE106382 and GSE20589. (A) PCA plot with GEOexplorer after removing batch effect. Points in the red boxes are GSE20589. (B) PCA plot by batches with RNAcare with log transformation after removing batch effect. We can see RNAcare provides a visualization by batches and can easily support combining more than 2 datasets. (C) Record comparison of GEOexplorer after batch correction.

```python
import requests
login_url='http://130.209.125.25:8000/accounts/rest/login/'
login_data={
    'username':'mt229a',
    'password':'password'
}
headers={'X-Requested-With':'XMLHttpRequest','Content-Type':'application/json'}
response=requests.post(login_url,headers=headers, json=login_data)
if response.status_code==200:
    token=response.json().get('token')
    print(f'Token: {token}')
else:
    print('Login failed: ',response.json())
```

```
Token: eyJ0eXAiOiJKV1QiLCJhbGciOiJIUzI1NiJ9.eyJ1c2VybmFtZSI6Im10MjI5YSIsImV4cCI6MTcyMzkyNTYyNH0.UnOwd6TcZvp5TVpYSGp3
J4VeI1qY-wCzIZPIZ99pYUc
```

```python
import requests
url='http://130.209.125.25:8000/'
headers={
    'Authorization':f'Bearer {token}',
}
response=requests.get(url,headers=headers)
print('Response Status Code:', response.status_code)
print('Response Text:', response.text)
```

```
Response Status Code: 200
Response Text: <!DOCTYPE html>

<html>
<head>
<style>
th,td{
padding:15px;
}
.table-condensed{
font-size: 10px;
}
.question{
max-width: 0.8%;
width:auto;
display: inline-block;
}
.ui-tooltip-content{
font-size:15px;
}
div.ui-tooltip{
max-width:800px;
max-height:800px;
}
.question{
content:url('/static/questionMark.png');
}
.errorlist{
            color: red;  /* Set the color of the error messages to red */
}
[illegible]
```

Supplemental Figure 3. We simulate requests from clients, firstly, the client requests a token using an existing username and password, after getting the token, then the client can access the resources with the URLs

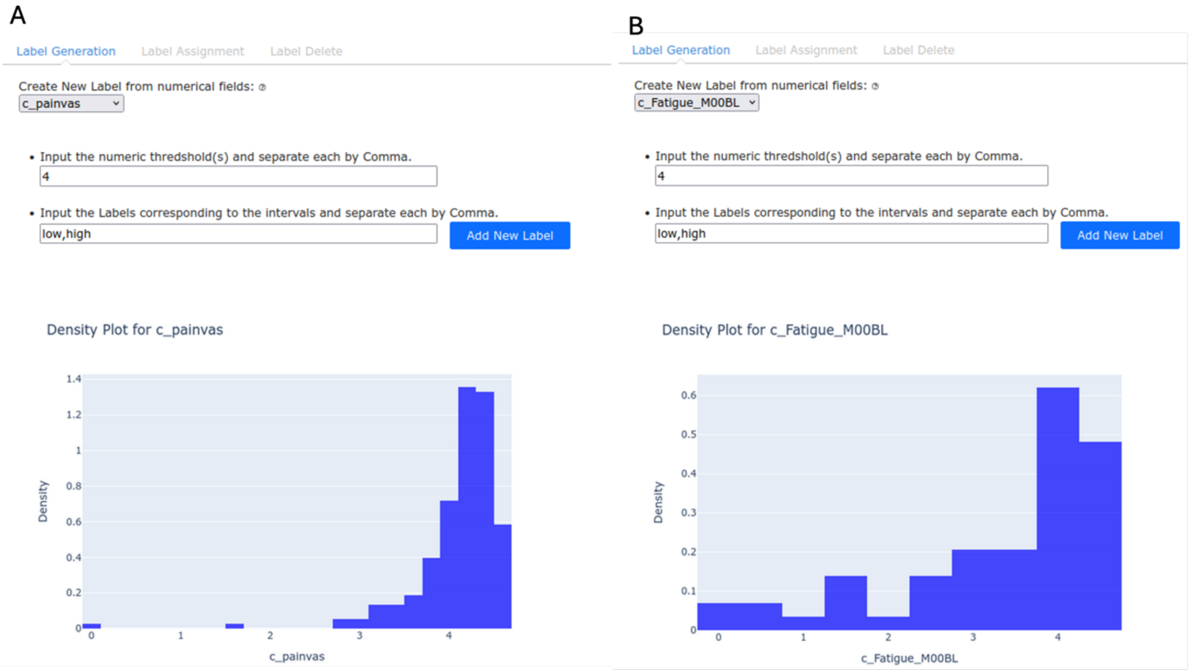

Supplemental Figure 4. (A) Integration of PEAC and ORBIT with label generation for pain VAS. (B) Label generation for Fatigue (RA-MAP)

Application		GenePattern Chapman et al.,2006 [50]	GEO2R Barrett et al., 2013 [51]	BicOverlapper 2 Santamaria et al., 2014 [52]	Babelomics Alonso et al., 2015 [53]	Morpheus Gould, 2016 [54]	START Nelson et al., 2017 [8]	BioJupies Torre et al., 2018 [55]	iDEP Ge et al., 2018 [6]	GREIN Mahi et al., 2019 [7]	EXPANDER Hait et al. 2019 [56]	RaNa-seq Prieto and Barrios, 2019 [57]	RNAdetector La Ferlita et al., 2021 [58]	Degust Powell, 2019 [59]	GEOexplorer Hunt et al., 2022 [12]	Phantasus, 2024 [9]	RNAcare
Gene expression analysis key steps	Normalization	+	-	-	+	+	+	+	+	+	+	-	-	-	+	+	+
	PCA	+	-	-	+	-	+	+	+	+	+	+	-	+	+	+	+
	Clustering	+	-	-	+	+	-	+	+	-	+	-	-	-	+	+	+
	Differential Expression	+	+	+	+	-	+	+	+	+	+	+	+	+	+	+	+
	Pathway analysis	+	-	+	+	-	-	+	+	-	+	+	-	-	+	+	+
	Batch integration	-	-	-	-	-	-	-	-	-	-	-	-	-	only two	-	+
Gene expression sources	user-provided data	+	-	+	+	+	+	+	+	-	+	+	+	+	-	+	+
	GEO microarray	+	+	+	-	-	-	-	-	-	-	-	-	-	+	+	Upload Manually/Automation under development
	GEO RNA-seq	-	-	-	-	-	-	Under development	Under development	+	-	+	-	-	-	Under development	Upload Manually/Automation under development
User experience features	Architecture	Web non-Shiny	Web non-Shiny	Local installation	Web non-Shiny	Web non-Shiny	Web Shiny	Web non-Shiny	Web Shiny	Web Shiny	Local installation	Web non-Shiny	Local installation	Web non-Shiny	Web Shiny	Web non-Shiny	Web Service/ Local installation
	Saved sessions	+	-	-	-	+/-*	-	+	-	-	-	+	-	-	-	+	-
	Interactive heatmap	-	-	+/-*	-	+	+/-*	+/-*	+/-*	+	+	+/-*	-	+	+	+	+
	Interactive plots	-	-	-	+	+	+	+	+	+	+	+	-	+	+	+	+
	Editing sample annotations	-	-	-	+	+	-	-	-	-	+	-	+	-	-	+	+
	Editing gene annotations	-	-	-	+	-	-	-	+	-	-	-	-	-	-	+	+

Supplemental Table 1. Comparison of different platforms. we consider existing software platforms for gene expression analysis. Although they have common characteristics, their implementations vary a lot, which affects their usability, scalability and robustness. For the comparison, we consider three aspects, repeating what Kleverov et al. 2024 [9] did: (1) support for gene expression analysis steps, (2) data availability and (3) user experience.

+/-* As processed in ARCHs4 and/or Dee2 projects.

Clinical fields \ Cohorts	PEAC	ORBIT	RA-MAP
abnormal		c_abn	
age	c_age	c_age	c_age
aggregated VAS score		c_agvas	
bmi			c_bmi
crp	c_crp	c_crp	c_crp
disease activity score	c_das	c_das	c_das
eq5d		c_eq5d	
eq5d VAS		c_eq5dvas	
esr	c_esr	c_esr	c_esr
fatigue VAS			c_fatigue
hospital anxiety and depression scale-anxiety subscale		c_hada	
categorical classification of the HADS-A score		c_hadacat	
categorical classification of the hospital anxiety and depression scale-depression subscale		c_haddcat	
haemoglobin			c_haemoglobin
health Assessment Questionnaire	c_haq	c_haq	
pain VAS	c_painvas	c_painvas	c_painvas
platelets			c_platelets
sex	c_sex	c_sex	c_sex
swollen joint count		c_sjc	
smoking status			c_smoke
total joint count		c_tjc	
white blood cells			c_whitebloodcells

Supplemental Table 2. Clinical fields for datasets used in the paper

Additional references:

50. Chapman SJ, Khor CC, Vannberg FO, Maskell NA, Davies CW, Hedley EL, et al. GenePattern 2.0. 2006.
51. Barrett T, Wilhite SE, Ledoux P, Evangelista C, Kim IF, Tomashevsky M, et al. NCBI GEO: Archive for functional genomics data sets - Update. Nucleic Acids Res. 2013;41.
52. Santamaría R, Therón R, Quintales L. BicOverlapper 2.0: Visual analysis for gene expression. Bioinformatics. 2014;30:1785–6.
53. Alonso R, Salavert F, Garcia-Garcia F, Carbonell-Caballero J, Bleda M, Garcia-Alonso L, et al. Babelomics 5.0: Functional interpretation for new generations of genomic data. Nucleic Acids Res. 2015;43:W117–21.
54. Gould J. Morpheus: Versatile matrix visualization and analysis. https://software.broadinstitute.org/morpheus/index.html. 2016.
55. Torre D, Lachmann A, Ma'ayan A. BioJupies: Automated Generation of Interactive Notebooks for RNA-Seq Data Analysis in the Cloud. Cell Syst. 2018;7:556-561.e3.
56. Hait TA, Maron-Katz A, Sagir D, Amar D, Ulitsky I, Linhart C, et al. The EXPANDER Integrated Platform for Transcriptome Analysis. J Mol Biol. 2019;431:2398–406.

57. Prieto C, Barrios D. RaNA-Seq: Interactive RNA-Seq analysis from FASTQ files to functional analysis. Bioinformatics. 2020;36:1955–6.
58. La Ferlita A, Alaimo S, Di Bella S, Martorana E, Laliotis GI, Bertoni F, et al. RNAdetector: a free user-friendly stand-alone and cloud-based system for RNA-Seq data analysis. BMC Bioinformatics. 2021;22.
59. David R. Powell. Degust: interactive RNA-seq analysis, DOI: 10.5281/zenodo.3258932.
